# Prevention of Carcinogenesis and Development of Gastric and Colon Cancers by 2-Aminophenoxazine-3-one (Phx-3): Direct and Indirect Anti-Cancer Activity of Phx-3

**DOI:** 10.3390/ijms140917573

**Published:** 2013-08-28

**Authors:** Akio Tomoda, Keisuke Miyazawa, Takafumi Tabuchi

**Affiliations:** 1 Department of Biochemistry, Tokyo Medical University, 6-1-1 Shinjuku, Tokyo 160-8402, Japan; E-Mail: miyazawa@tokyo-med.ac.jp; 2 Fourth Department of Surgery, Tokyo Medical University, Ibaragi Medical Center, 3-20-1 Chuo, Ami, Inashiki, Ibaraki 300-0395, Japan; E-Mail: tabuchi@tokyo-med.ac.jp

**Keywords:** Phx-3, gastric and colon cancers, apoptosis, neutrophils

## Abstract

2-Aminophenoxazine-3-one (Phx-3), an oxidative phenoxazine, exerts strong anticancer effects on various cancer cell lines originating from different organs, *in vitro*. This article reviews new aspects for the prevention of carcinogenesis and development of gastric and colon cancers by Phx-3, based on the strong anticancer effects of Phx-3 on gastric and colon cancer cell lines (direct anticancer effects of Phx-3 for preventing development of cancer), the bacteriocidal effects of Phx-3 against *Helicobacter pylori* associated with carcinogenesis of gastric cancer (indirect anticancer effects for preventing carcinogenesis of gastric cancer), and the proapoptotic activity of Phx-3 against human neutrophils involved in the incidence of ulcerative colitis associated with a high colon cancer risk (indirect anticancer effects for preventing carcinogenesis of colon cancer).

## 1. Introduction

**Figure 1:**
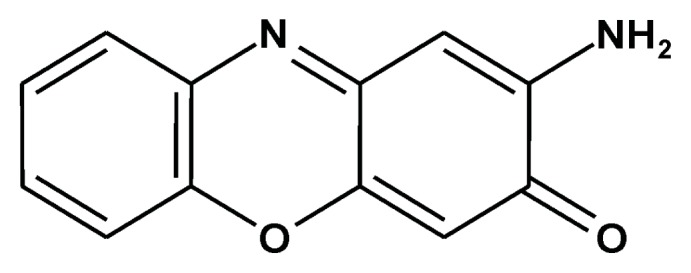
Chemical structure of Phx-3.

2-Aminophenoxazine-3-one (Phx-3) (Figure 1) was first reported as the antibiotics for streptomyces discovered in soil in Kunitachi, Tokyo, Japan, in 1959 [1]. However, no further characterization of its bioactivity was carried out, possibly due to its weaker (and questionable) antibiotic effects against various microbes except for *Mycobacterium tuberculosis*, and therefore, it was named questiomycin. Tomoda and colleagues found in 1986 [2] that Phx-3 can be produced during the reaction of human erythrocytes or hemoglobin with *o*-aminophenol, and later demonstrated that it does exert strong anticancer effects both on various cancer cell lines [3–8] and cancer cell-transplanted mice *in vivo* [9,10], as well as antimicrobial effects against *Helicobacter pylori* [11], *Chlamydia pneumoniae* [12], some mycobacterial species [13], and herpes viruses [14]. Their studies demonstrated that Phx-3 causes apoptotic cell death in the gastric and colon cancer cell line by decreasing intracellular pH, dysregulating the function of mitochondria, and activating the caspase signaling [8,15–17]. Phx-3 also efficiently suppresses the activity of Akt, which is responsible for the cell survival of cancer cells [7]. These results indicate that Phx-3 has the potential to promote apoptosis of cancer cells, and therefore, may be applied to treat such cancers as gastric cancer and colon cancer, which are refractory to chemotherapy. This article describes the anticancer activity of Phx-3 against gastric and colon cancer cells in terms of preventing the development of gastric and colon cancers (direct effects of Phx-3).

Recent research indicates that inflammation is a critical component of tumor development [18–20], and that neutrophils, which play an important role in the inflammatory reactions in the body, are potentially involved in the carcinogenesis, metastasis and angiogenesis of cancers [21]. In 1992, Tabuchi *et al*. [22] found that tumor size significantly reduced when neutrophil (=granulocyte) was deleted by extracorporeal circulation after being transplanted with VX2 carcinoma cells, a rabbit papilloma cell line. Later, they demonstrated that neutrophils play a critical role in the development of colon cancer. Namely, using granulocyte apheresis to remove these cells from the blood of patients with colon cancer extensively reduced the size of the cancer, and they proposed that the granulocytes/lymphocytes (G/L) ratio might be pivotal to the development of colon cancer in the body [23,24]. This view has been extensively supported by reports that the increased number of neutrophils in patients with colon cancer is evident [25] and that the G/L ratio in the peripheral blood is significantly higher in the advanced stages of colon cancer [26,27]. In addition, neutrophils that produce a large amount of active oxygen during inflammation are involved in ulcerative colitis and causative of the carcinogenesis of colon cancer [28,29]. Therefore, suppression of the activity of neutrophils may lead to preventing ulcerative colitis, and finally to preventing the carcinogenesis of colon cancer. Recently, Tabuchi *et al*. [30] found that Phx-3 exerted a strong proapoptotic effect on neutrophils in human blood and almost completely suppressed the generation of superoxide (O_2_^−^) from human neutrophils. Kohno *et al*. [31] also indicated that Phx-3 has the ability to suppress inflammation, because Phx-3 inhibited the activity of cyclooxygenase-1 (Cox-1) and Cox-2, that of nitric oxide synthetase, and the production of interleukin 6 in macrophages, which are significantly involved in inflammation. Thus, we assumed that the anticancer activity of Phx-3 may be exerted through the suppression of superoxide generation and reduction in the number of neutrophils in the peripheral blood in patients with colon cancer, which may be designated as an indirect anticancer activity of Phx-3 for preventing carcinogenesis of colon cancer.

Moreover, *H. pylori* is a major factor in the carcinogenesis of gastric cancer including gastric lymphoma and adenocarcinoma, and abolishing these bacteria from the stomach may reduce the incidence of the gastric cancer [32,33]. Hanawa *et al*. [11] found that Phx-3 efficiently kills *H. pylori*, *in vitro*, which should be regarded as an indirect anticancer activity of Phx-3 on gastric cancer carcinogenesis. These indirect anticancer effects of Phx-3 were analyzed in terms of preventing carcinogenesis of gastric and colon cancers.

## 2. Direct Anticancer Activity of Phx-3 on Gastric and Colon Cancers

Gastric cancer and colon cancer, mostly adenocarcinoma, are refractory to chemotherapy alone [34,35]. Gastric cancer is a common malignancy in Japanese adults and has recently increased in Western adults [34,36]. Colon cancer is a major public health problem worldwide, and its incidence is increasing annually [35,37]. In spite of tremendous efforts to prevent the development of gastric and colon cancers, the anticancer drugs to abolish these malignancies are not still developed; thus, surgical therapy is a first-line treatment of these types of cancer. Since we found that Phx-3 extensively prevents the growth of gastric and colon cancer cell lines *in vitro* [8,15–17], we analyzed its cytotoxic and proapoptotic activities, focusing on the anticancer mechanism for preventing the development of gastric and colon cancer.

### 2.1. Cytotoxic and Proapoptotic Effects of Phx-3 on Gastric and Colon Cancer Cells *in Vitro*

Kasuga *et al*. [15] examined the anticancer activity of Phx-3 on human gastric cancer cell lines, MKN45, MKN74, MKN7 and KATO III cells *in vitro*, and demonstrated that Phx-3 caused cytotoxic (50% inhibition of cell growth, IC_50_: less than 10 μM) and proapoptotic effects on these cells at lower concentrations. These cytotoxic effects of Phx-3 were stronger than those of 5-fluorouracil that is clinically administered to patients with the advanced gastric cancer, taking into account the results of Osaki *et al*. [38] that 1 mM 5-fluorouracil induced variable degrees of apoptosis in the cultured gastric cancer cell lines (14% in MKN74, 12% in MKN45, 3% in MKN28 and 0.5% in KATO-III), and 50 μM 5-fluorouracil had little effect on the induction of apoptosis in these gastric cancer cell lines. In addition, Nakachi *et al*. [17] studied the anticancer activity of Phx-3 on human colon cancer cell lines, COLO20, DLDl and PMCO1 cells, and demonstrated that it caused cytotoxic (IC_50_: less than 10 μM) and proapoptotic effects on these cells. Cell growth was extensively inhibited by Phx-3 in another colon cancer cell line, Lovo-1 (IC_50_: 20 μM), as well [8]. These results indicate that Phx-3 exhibits strong anticancer activity against gastric and colon cancer cells, *in vitro*. Phx-3 did not affect the growth of the normal cell line—human umbilical vein endothelial cells (HUVECs)—at concentrations less than 60 μM [39], suggesting that Phx-3 may exert a less adverse effect on normal cells.

### 2.2. Phx-3 Induces the Apoptotic Cell Death of Gastric and Colon Cancer Cells by Reducing the Higher pHi of These Cells

Extracellular pH (pHe) is usually higher than pHi in normal cells, as explained by Donnan’s classical membrane equilibrium theory. However, this view does not necessarily explain the extremely acidic pH in the outer interstitial medium of tumor tissues [40–42]. Therefore, it has been assumed that some mechanism to discharge the hydrogen ion from the cytosol to the extracellular medium might operate in cancer cells. Recent findings indicate that Na^+^/H^+^ exchanger isoform 1 (NHE1), which is ubiquitously present in the plasma membrane in normal and cancerous cells, plays a pivotal role in regulating pHi in cancer cells by discharging the cytosolic hydrogen ion to the outside [43–45]. Actually, pHi is more alkaline in cancer cells than in normal cells. Reshkin *et al*. [46] reported that pHi is far higher in cancer cells (7.12–7.7) than in normal cells (6.99–7.05). Che *et al*. [8,47] demonstrated that pHi in more than 10 species of cancer cell lines originating from different organs exceeded 7.4 (pHi in the extreme case was 7.7).

Nagata *et al*. [16] found that pHi in the human gastric cancer cell lines MKN45 and MKN74 exceeds pHe (pHi of 7.48 for MKN45 and 7.5 for MKN74 cells, *versus* medium pH of 7.4). Colon cancer cell line, Lovo-1 cells, exhibited higher pHi (pHi = 7.61) [7]. Higher pHi in cancer cells seems to be suitable for proliferation and oncogene transformation of the cells [48,49], and increases tumorigenesis [48–51]. Therefore, agents to reduce pHi in cancer cells might be promising anticancer drugs, as has been predicted by several researchers [40,46,50]. Nagata *et al*. [16] demonstrated that when human gastric cancer cells (including MKN45 and MKN74 cells) were treated with Phx-3, pHi decreased rapidly, dependent on the dose of Phx-3, and such intracellular acidification was sustained for at least 4h. In particular, pHi decreased by 1.6pH units in MKN45 cells and 1.2pH units in MKN74 cells immediately after administration of 100 μM Phx-3. Such rapid and drastic changes in pHi in these gastric cancer cells could be explained by the inhibition of NHE1 caused by administering Phx-3. A close relationship between anticancer activity of Phx-3 on these gastric cell lines and a decreased extent of pHi caused by Phx-3 was shown [16]. It also confirmed a causal relationship between ΔpHi and cytotoxic effects due to Phx-3 in various cancer cell lines [8].

An extensive pHi decrease may promote proapoptotic signaling in gastric and colon cancer cells. Specifically, the decrease of pHi activates caspase-3, an important executor of apoptosis, and DNase II, a pH-dependent endonuclease that is responsible for DNA fragmentation, thus inducing apoptotic cell death of these cancer cells [52,53]. This view is consistent with a report by Perez-Sala *et al*. [54] that when HL-60 cells (a human leukemia cell line) were treated with either ionomycin or lovastatin, cellular apoptosis was induced as a result of intracellular acidification and activation of DNase II.

The mechanism by which Phx-3 inhibits NHE1 may be explained by the findings of Hendrich *et al*. [55] that phenoxazine molecules are located close to the polar/apolar interface of lipid bilayers and weakly interact with lipid bilayers, altering the lipid phase properties of the cellular membranes. It is possible that rapid changes in the conditions of the plasma membranes induced by Phx-3 significantly affect the activity of NHE1 in the cellular membranes. It is noteworthy that the action of Phx-3 seems to be opposite to that of 12-*O*-tetradecanoylphorbol-13-acetate (TPA), a strong cancer promoter that interacts with phospholipid bilayers to increase pHi. This view is consistent with the findings of Azuine *et al*. [56] that the cancer promotion action of TPA was cancelled by the phenoxazine compounds.

### 2.3. Dysfunction of the Mitochondria in Gastric and Colon Cancer Cells by Phx-3

Depolarization of mitochondria is an eminent change that activates caspases and greatly influences the apoptotic events in cancer cells [52,53]. In gastric and colon cancer cells, treatment with Phx-3 significantly depolarizes mitochondria, followed by apoptotic cell death of these cancer cells [8,16]. This result is consistent with recent reports [6,8] that Phx-3 depolarizes mitochondria in a variety of cancer cell lines originating from different organs. Therefore, Phx-3 may exert strong anticancer activity on gastric and colon cancers via depolarization of mitochondria in these cells.

### 2.4. Plausible Mechanism for the Anticancer Effects of Phx-3 on Gastric and Colon Cancer Cells

**Scheme 1:**
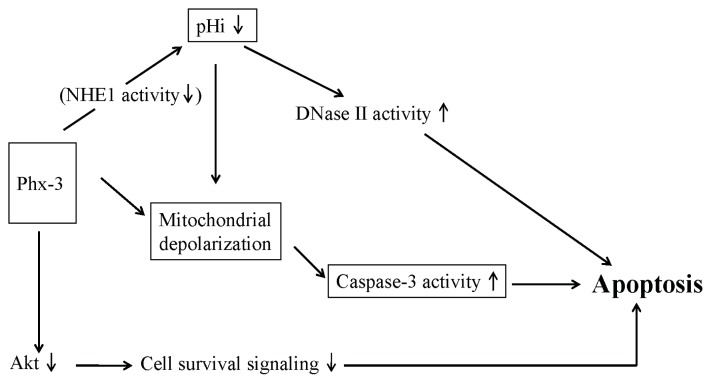
Plausible mechanism for anticancer effects of Phx-3 on gastric and colon cancer cells.

The proapoptotic activity of Phx-3 may be explained by the mechanism depicted in Scheme 1. First, Phx-3 may inhibit the activity of NHE1 and cause a drastic reduction of pHi in gastric and colon cancer cells, which activates caspase-3 and DNase II that are responsible for apoptotic cell death signaling in these cells.

Serine/threonine kinase Akt mediates a variety of survival signaling, participating in the growth factor maintenance of cell survival and preventing cancer cells from becoming apoptotic. Therefore, it is important to suppress Akt signaling to prevent the survival of cancer cells, and agents to inhibit activity of Akt may be promising for cancer chemotherapy. Enoki *et al*. [57] initially reported the significant activity of Phx-1 (2-amino-4,4α-dihydro-4α,7-dimethyl-3H-phenoxazine-3-one), another oxidative phenoxazine, in suppressing the phosphorylation of Akt in rat basophilic leukemia RBL-2H3 cells. Hara *et al*. [58] demonstrated that Phx-1 inhibits the proliferation and serum-induced phosphorylation of Akt in Jurkat cells, a human T cell leukemic cell line. Thimmaiah *et al*. [59] examined in detail the effect of various synthetic phenoxazines and demonstrated that *N*^10^-substituted phenoxazines such as 10-[4′-(*N*-diethylamino) butyl]-2-chlorophenoxazine and 10-[4′ ((beta-hydroxyethyl)piperazineo)-butyo]-2-chlorophenoxazine strongly inhibited Akt phosphorylation. Zheng *et al*. [7] demonstrated that Phx-3 inhibited the phosphorylation of Akt in human lung adenocarcinoma cell line A549 cells, suggesting that Phx-3 strongly suppresses Akt signaling in cancer cells, though it is not clear whether Phx-3 inhibits Akt signaling in gastric and colon cancer cells, therefore being still hypothetical. Based on these results, Scheme 1 summarizes the plausible mechanism for Phx-3 inducing apoptosis in cancer cells.

## 3. Indirect Anticancer Activity of Phx-3 on Gastric and Colon Cancers

*H. pylori* infection is associated with gastric lymphomas and adenocarcinomas, and now is designated as class I carcinogens [32,33]. Therefore, eliminating these bacteria would prevent carcinogenesis of gastric cancer. Hanawa *et al*. [11] reported that *H. pylori* is sensitive to Phx-3 treatment at a lower concentration of 2 μM, suggesting that Phx-3 may contribute to preventing carcinogenesis of gastric cancer by killing these bacteria. Overexpression of Cox-2 increases the proliferation of gastric cancer cell lines, and inhibiting Cox-2 slows the growth of stomach cancer xenografts in nude mice [60]. Kohno *et al*. [31] demonstrated that Phx-3 inhibits Cox-2 in macrophages. Thus, Phx-3 may slow the growth of stomach cancer.

Neutrophils have recently attracted much attention in terms of carcinogenesis, metastasis and angiogenesis of cancers. According to a recent review by Gregori and Houghton [21], neutrophils influence inflammatory cell recruitment and activation by producing cytokines and chemokines, and regulate tumor cell proliferation, angiogenesis, and metastasis by secreting reactive oxygen species (ROS) and proteases. Thus, the manipulating neutrophils may be useful for treating cancers. In 1995, Tabuchi *et al*. [23] first indicated that neutrophils play a critical role in the development of colon cancer, because using granulocyte apheresis to remove these cells from the blood of patients with colon cancer extensively reduced the cancer size. Shimazaki *et al*. [61] also indicated that the G/L ratio is clinically relevant biomarker of long-term cancer progression in patients with colorectal cancer. Therefore, drugs that selectively decrease the number of circulating neutrophils in the blood may be beneficial for treating colon cancer. The recent discovery of Tabuchi *et al*. [30] that when freshly obtained blood was treated with 50 μM Phx-3 at 37 °C for 18 h, the population of apoptotic neutrophils increased to more than 50%, but no apoptotic lymphocytes were seen, may be of significance in treating colon cancer. This would be designated as an indirect anticancer effect of Phx-3 on colon cancer.

Neutrophils seem to be involved in ulcerative colitis, which is associated with the carcinogenesis of colon cancer [28,29]. Neutrophils, which produce a large amount of active oxygen during inflammation [62,63], may be associated with the onset of ulcerative colitis [28,29], therefore, suppression of neutrophil activity may lead to preventing ulcerative colitis, and finally to preventing the carcinogenesis of colon cancer. Phx-3 may be a promising drug for treating ulcerative colitis because it almost completely suppressed generation of superoxide (O_2_^−^) from human neutrophils *in vitro* [30]. Such indirect anticancer effects of Phx-3 on carcinogenesis of colon cancer should be confirmed by further experiments using animals.

## 4. Future Aspect of Phx-3 as Anticancer Drug for the Treatment of Gastric and Colon Cancers

In this review, we surveyed the direct and indirect anticancer activity of Phx-3 on gastric and colon cancer; however, these findings should be confirmed in experiments using animals. Though we did not apply Phx-3 to mice with gastric or colon cancer, we found that Phx-3 extensively suppressed the development and metastasis of malignant melanoma cells transplanted in mice [9,10]. The results of the panel test conducted by Miyake *et al*. [64] may suggest the availability of Phx-3 to humans, because they demonstrated that when the patients with gastritis were administered with the tablet (200 mg) containing 1 μg Phx-3 after each dinner for one week, their gastric conditions were much improved, as evaluated based on the patients’ conditions of the stomach, in comparison with the control patients without administration of the tablet. Also, Phx-3 had few adverse effects on mice at higher doses [9,10]. Kohno *et al*. [31] reported that oral administration of 500–1500 mg/kg Phx-3 to ddY mice did not cause gastrointestinal injury and that repeated oral administration of 10 mg/kg Phx-3 to mice for four weeks caused no diarrhea. Therefore, it would be significant to investigate whether or not Phx-3 holds promise as an agent to treat human gastric and colon cancer.
